# Exercise Alleviates the Apolipoprotein A5-Toll-Like Receptor 4 Axis Impairment in Mice With High-Fat Diet-Induced Non-alcoholic Steatohepatitis

**DOI:** 10.3389/fphys.2021.783341

**Published:** 2021-12-31

**Authors:** Yang Yu, Lina Yu, Nuo Cheng, Xiaoguang Liu, Chunlu Fang, Shujing Liu, Lin Zhu

**Affiliations:** ^1^Department of Sports and Health, Guangzhou Sport University, Guangzhou, China; ^2^Department of Preventive Dentistry, Affiliated Stomatology Hospital of Guangzhou Medical University, Guangdong Engineering Research Center of Oral Restoration and Reconstruction, Guangzhou Key Laboratory of Basic and Applied Research of Oral Regenerative Medicine, Guangzhou, China; ^3^Graduate School of Guangzhou Sport University, Guangzhou, China; ^4^Center for Scientific Research and Institute of Exercise and Health, Guangzhou Sport University, Guangzhou, China; ^5^Guangdong Provincial Key Laboratory of Physical Activity and Health Promotion, Guangzhou Sport University, Guangzhou, China

**Keywords:** exercise, ApoA5, TLR4, LPS, non-alcoholic steatohepatitis (NASH)

## Abstract

**Background:** Apolipoprotein A5 (ApoA5), an important modulator of plasma and hepatic triglyceride metabolism, has been found to be downregulated by metformin to improve non-alcoholic fatty liver disease. Meanwhile, exercise has been recommended as a therapeutic strategy for non-alcoholic steatohepatitis (NASH). However, no study has yet determined whether exercise affects hepatic ApoA5 expression or the inhibition of ApoA5 to toll-like receptor 4 (TLR4). We herein examined the effects of exercise on hepatic ApoA5 expression and the relevance of ApoA5 and TLR4-mediated pathway in mice with high-fat diet (HFD)-induced NASH.

**Methods:** Male C57BL/6J mice were built NASH model with high-fat diet for 12 weeks, and following mice were subjected to exercise for 12 weeks on a treadmill. Microscopy and enzyme-linked immunosorbent assay were used to measure histological analysis of liver and hepatic lipids, respectively. Quantitative real-time PCR and western blot were used to determined mRNA and protein levels of ApoA5 and TLR4-mediated nuclear factor kappa B (NF-κB) pathway components, respectively. ApoA5 overexpression plasmids transfected into mice to investigate the relevance of ApoA5 and TLR4.

**Results:** 12 weeks of exercise remarkably alleviated HFD-induced hepatic lipid accumulation, inflammation, and fibrosis, as well as reduced serum lipopolysaccharide (LPS), hepatic TLR4, myeloid differentiation factor 88 (MyD88), and NF-κBp65 expression. Importantly, exercise did not reduce ApoA5 expression but instead enhanced its ability to suppress TLR4-mediated NF-κB pathway components by decreasing circulating LPS in our experiments involving transfection of ApoA5 overexpression plasmids and LPS interventions.

**Conclusion:** The results demonstrated that exercise improved HFD-induced NASH by triggering the inhibitory effects of ApoA5 on the TLR4-mediated NF-κB pathway.

## Introduction

Non-alcoholic steatohepatitis (NASH) is a severe manifestation of non-alcoholic fatty liver disease (NAFLD) and has been considered the main cause of liver failure, cirrhosis, and cancer (Todoric et al., 2020). The lipid metabolic disorder is an important pathogenesis factor of NASH (Yu Y. et al., 2019). Reports have shown that more than 20% of patients with NASH will have developed cirrhosis during their lifetime (Younossi et al., 2018). Regrettably, no specific drugs have yet been approved for NASH, making liver transplantation the leading treatment method in recent years (Noureddin et al., 2018). At present, lifestyle modifications, such as exercise, have been primarily recommended for the prevention and treatment of NASH in the United States, Europe, and China (Fan and Farrell, 2009; Chalasani et al., 2012; European Association for the Study of the Liver et al., 2016). However, the mechanisms responsible for the protective effects of exercise against NASH have remained unclear.

Apolipoprotein A5 (ApoA5), a member of the apolipoprotein family specifically expressed in the liver (O’Brien et al., 2005), has been considered as an important modulator of plasma and hepatic triglyceride (TG) metabolism in earlier studies (Pennacchio et al., 2001; Priore Oliva et al., 2005; Shu et al., 2007; Shu et al., 2010; Sharma et al., 2013). Shu et al. (2010) and Blade et al. (2011) showed that *APOA5* transgenic mice and hepatoma cells transfected with ApoA5 expression plasmids exhibited increased hepatic TG accumulation. Furthermore, recent data have indicated that hepatic ApoA5 mRNA and protein is overexpressed in patients and mice with NAFLD. The hepatic steatosis and other phenotypes of NAFLD may be alleviate with decrease of ApoA5 mRNA expression or with down-regulation of ApoA5 involving signaling pathway (Ress et al., 2011; Feng et al., 2015; Lin et al., 2017). But there was an opposite result. van den Berg et al. (2013) showed that *APOA5* (−/−) mice fed high fat diet manifest greater hepatic steatosis, and ApoA5 overexpression prevented ectopic lipid accumulation rather than increasing it. These findings implicated that ApoA5 may be as a potential therapeutic target for NASH, whereas the mechanisms need to be clarified. Additionally, ApoA5 acts as a predictor for remnant liver growth after preoperative portal vein embolization and liver surgery (Hoekstra et al., 2012). Evidence has shown that hepatic ApoA5 overexpression inhibited the protein expressions of toll-like receptor 4 (TLR4) and TLR4-mediated signaling pathway, thereby alleviating fulminant liver failure in mice (Tao et al., 2019). However, the inhibitory effects would weaken with increasing concentrations of lipopolysaccharide (LPS) despite ApoA5 overexpression (Tao et al., 2019).

Lipopolysaccharide is part of the outer membranes of gram-negative bacteria, with its circulating concentrations significantly increasing in mice with high-fat diet (HFD)-induced NASH (Aron-Wisnewsky et al., 2020). However, LPS was partially reduced after exercise (Yu C. et al., 2019). Moreover, mRNA and protein expression of ApoA5 was remarkably increased in mice with HFD-induced NASH (Ress et al., 2011; Feng et al., 2015; Lin et al., 2017), implying that the inhibitory effects of ApoA5 on TLR4-mediated signaling pathway depended on the reduction of circulating LPS concentrations in mice with HFD-induced NASH. This study therefore established a HFD-induced NASH model to investigate the effects of exercise on hepatic ApoA5 expression and determine the relevant of ApoA5 and TLR4-mediated signaling pathway.

## Materials and Methods

### Animal Model

Male C57BL/6J mice aged 6 weeks were purchased from the Experimental Animal Center of Guangdong Province (Guangzhou, China) and acclimated for 1 week. The mice were housed on a 12-h light–dark cycle at 22–24°C and were provided free access to food and water. All animal care and lab experimental procedures were conducted in accordance with the Chinese Guidelines for Animal Welfare and Experimental Protocols and were approved by the Animal Experiment Administration Committee of Guangzhou Sport University (2020DWLL-005). All mice were randomly divided into three groups: low-fat diet control group (LFD, *n* = 24), a high-fat diet group (HFD, *n* = 24), and a high-fat diet plus exercise group (HFD + EXE, *n* = 24). The LFD group received a low-fat diet containing 10% kcal from fat (D12450J, Research Diets Inc.), whereas the HFD and HFD + EXE groups were fed a HFD containing 60% kcal from fat (D12492, Research Diets Inc.) for 24 weeks, and after 12 weeks of HFD feeding, mice in HFD + EXE group were subjected to exercise training for 12 weeks. A day after the final training session, mice were killed under anesthesia (sodium pentobarbital 50 μg/g) for collection of serum and liver.

### Training Procedures

The exercise group was trained at 0% grade 5 days per week for 12 weeks on a treadmill. After a 5 min of warm up period at 6 m/min, mice performed 20 min of main exercise at 10 m/min and 5 min of cool down at 6 m/min were performed during the first week for adaptation. From the 2nd week to the 12th week, mice performed 5 min of warm up at 6 m/min, 50 min of main exercise at 12 m/min (75% maximum oxygen consumption) (Fernando et al., 1993), and 5 min of cool down at 6 m/min were performed.

### Hepatic Triglyceride and Total Cholesterol Analysis

Hepatic TG and TC levels were measured using commercial kits (Jiancheng Bioengineering Institute, Nanjing, China), according to the manufacturer’s instructions.

### Histological Analysis of Liver

Fresh liver tissues were fixed with 4% paraformaldehyde solution for 24 h, embedded in paraffin, and sliced into 4-μm sections for hematoxylin-eosin (H&E) staining and Sirius Red staining. The NAFLD activity score (NAS) was calculated according to the guidance provided by the Pathology Committee of the NASH Clinical Research Network (Kleiner et al., 2005): steatosis (<5% = 0, 5–33% = 1, 33–66% = 2, >66% = 3), lobular inflammation (none = 0, <2 foci = 1, 2–4 foci = 2, >4 foci = 3), and hepatocellular ballooning (none = 0, few = 1, prominent = 2). All features were scored in a blinded manner based on six fields of view per sample. Individual scores for each field of view were summed to calculate the NAS for each animal. Histological assessments were performed by a pathologist who was blinded to the treatment.

### Blood Analysis

Serum lipopolysaccharide (LPS) levels was measured using ELISA kits (CUSABIO Technology LLC.), according to the manufacturer’s instructions.

### Examining the Effects of Apolipoprotein A5 on Toll-Like Receptor 4-Mediated Nuclear Factor Kappa B Pathway

To analyze the effects of ApoA5 on TLR4-mediated signaling pathway, LFD mice were randomly divided into three groups (*n* = 4/group) and injected with an ApoA5 overexpression plasmid (pEGF-N1-ApoA5, 10 μg), negative control empty vector (pEGF-N1 vector, 10 μg), and normal saline through the tail vein, respectively. The mice were killed, after which serum and liver samples were harvested and stored for analysis after treatment for 3 days. The ApoA5 overexpression plasmid (pEGF-N1-ApoA5) and negative control empty vector (pEGF-N1 vector) were designed and purchased from Heyuan Biotechnology (OBIO, China).

### Examining the Effect of Lipopolysaccharide on the Ability of Apolipoprotein A5 to Inhibit Toll-Like Receptor 4-Mediated Nuclear Factor Kappa B Pathway

To investigate the ability of ApoA5 to inhibit TLR4-mediated NF-κB pathway within a certain LPS concentration, HFD + EXE mice were randomly divided into three groups (*n* = 4/group) that subsequently received intraperitoneal injections of normal saline, 5 μg/kg⋅wt LPS and 10 μg/kg⋅wt LPS, respectively. After the mice were killed, serum and liver samples were harvested and stored for analysis after treatment for 12 h. LPS (*Escherichia coli*, 0111:B4) was purchased from Sigma (St. Louis, MO).

### Quantitative Real-Time PCR

The primer sequences used herein are detailed in Table 1. Expression levels were normalized to those of the housekeeping gene GAPDH.

**TABLE 1:** Primer sequences used for qRT-PCR.

Gene	Forward primer (5′–3′)	Reverse primer (5′–3′)
ApoA5	AGTTGGAGCAAAGGCGTGAT	TTTCCGAATGCCTTCTGGGT
TLR4	TGAGGACTGGGTGGAAATG AGC	CTGCCATGTTTGAGCAATCTCAT
MyD88	TGACCCCACTCGCAGTTTGT	TTTGTTTGTGGGACACTGCTTTC
NF-κB	CGAGTCTCCATGCAGCTACG	TTTCGGGTAGGCACAGCAATA
Collagen I	GCCCGAACCCCAAGGAAG AAGC	CTGGGAGGCCTCGGTGACA TTAG
GAPDH	CCTCGTCCCGTAGACAAAATG	TGAGGTCAATGAAGGGGTCGT

### Western Blot Analysis

Protein was extracted from mouse livers. Total protein concentrations were measured using a BCA protein assay kit (Thermo Fisher Scientific). Equal amounts of total protein were separated using sodium dodecyl-sulfate polyacrylamide gel electrophoresis and transferred to polyvinylidene fluoride membranes. These membranes were then blocked and incubated with primary antibodies against ApoA5 (Abcam, Cambridge, MA, United States, ab239579), TLR4, MyD88, NF-κBp65, and β-actin (Cell Signaling Technology, Beverly, MA, United States, #14358, #4283, #8242 and #4970). Membranes were incubated for 1 h with the following secondary antibodies: goat anti-mouse IgG-HRP and mouse anti-rabbit IgG-HRP (Cell Signaling Technology, Beverly, MA, United States, #43593 and #58802). Signal detection was performed using SuperSignal Dura Substrate (Pierce, Biotechnology, United States), after which immunoblot signals were quantified using Quantity One software.

### Statistical Analysis

All data were expressed as mean ± standard error of the mean. Statistical significance was evaluated using one-way analysis of variance with the Bonferroni test for multiple comparisons. All analyses were performed using GraphPad Prism 5.0, with a *P* value of ≤0.05 indicating statistical significance.

## Results

### Exercise Ameliorates High-Fat Diet-Induced Body and Liver Weight Gain, Hepatic Lipid Accumulation, Inflammation, and Fibrosis

**FIGURE 1:**
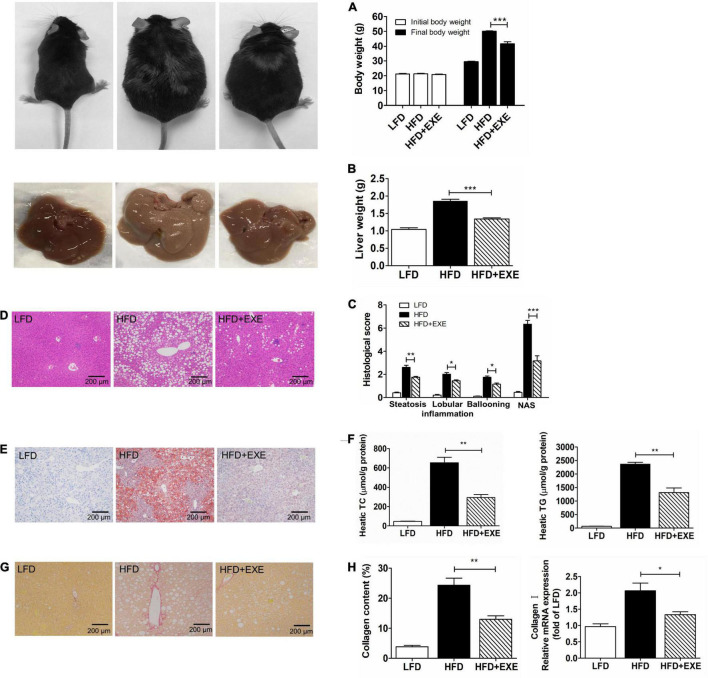
Exercise reduced HFD-induced body weight, liver weight, hepatic steatosis, inflammation, and fibrosis. The body weight **(A)** and liver weight **(B)** decreased after 12 weeks of exercise training. Liver sections stained with H&E (×100) **(C)**. Hepatic histological analysis of steatosis, inflammation, ballooning, and NAFLD activity score (NAS) **(D)**. Hepatic lipid accumulation as determined by Oil Red O staining (×100) **(E)**. Hepatic TC levels and TG levels **(F)**. Liver sections stained with Sirius Red (×100) **(G)**. Collagen content determined by counting Sirius Red positive areas in six randomly selected fields using Image Pro Plus 6.0 software and hepatic mRNA levels of Collagen I **(H)**. Results are presented as the mean ± SEM, *n* = 6–8 per group. * *P* < 0.05, ** *P* < 0.01, and *** *P* < 0.001.

Non-alcoholic steatohepatitis is characterized by hepatic steatosis, inflammation, and fibrosis (Tiniakos et al., 2010; Liu et al., 2016). Mice with HFD-induced NASH had increased body weight and liver weight, which reduced significantly after 12 weeks of exercise training (Figures 1A,B). The HFD + EXE group had significantly lower hepatic lipid accumulation, total cholesterol (TC), and TG levels compared to the HFD group (Figures 1E,F). Exercise significantly reduced histological parameters reflecting hepatic steatosis and inflammation, such as the steatosis score, lobular inflammation score, ballooning score, and total NAFLD activity score (NAS) (Figures 1C,D). Sirius Red staining showed that exercise suppressed HFD-induced collagen accumulation (Figures 1G,H). The HFD + EXE group had lower collagen I mRNA levels compared to the HFD group (Figure 1H). The aforementioned results indicated that 12 weeks of exercise training remarkably ameliorated HFD-induced NASH.

### Exercise Does Not Significantly Decrease Hepatic Apolipoprotein A5 Expression but Reduces Circulating Lipopolysaccharide Concentrations and Inhibits Hepatic Toll-Like Receptor 4-Mediated Signaling Pathway

**FIGURE 2:**
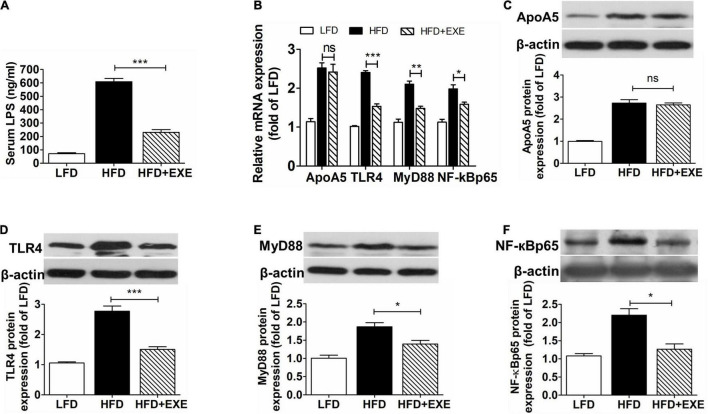
Exercise did not affect hepatic ApoA5 expression but reduced hepatic TLR4-mediated pathway components and circulating LPS concentrations. Circulating LPS concentrations **(A)**. Hepatic mRNA levels of ApoA5, TLR4, MyD88, and NF-κBp65 **(B)**. Western blot analyses of ApoA5 **(C)**, TLR4 **(D)**, MyD88 **(E)**, and NF-κBp65 **(F)**. Results are presented as the mean ± SEM, *n* = 4–6 per group. * *P* < 0.05, ** *P* < 0.01, *** *P* < 0.001, and *^ns^* *P* > 0.05 (no difference).

Compared to the LFD group, the HFD group exhibited significantly higher circulating LPS concentrations (609.42 ± 42.21 vs. 71.11 ± 14.15 ng/mL), which sharply declined after exercise (230.88 ± 35.03 vs. 609.42 ± 42.21 ng/mL) (Figure 2A). Although exercise slightly decreased ApoA5 mRNA and protein expression in the liver, no significant difference in hepatic mRNA and protein expression of ApoA5 was observed between the HFD and HFD + EXE groups (Figures 2B,C). Compared to the LFD group, HFD group exhibited higher levels of TLR4 (Figures 2B,D). Exercise reduced both mRNA and protein levels of TLR4 (Figures 2B,D). Moreover, MyD88 and NF-κBp65 mRNA and protein levels were also markedly lowered by exercise (Figures 2E,F). Supporting such findings, one study showed that high expression of ApoA5 can inhibit TLR4 expression (18). The aforementioned results implied that exercise enhanced the ability of ApoA5 to inhibit the TLR4-mediated signaling pathway at certain LPS concentrations.

### Exercise Enhances the Ability of Apolipoprotein A5 to Inhibit the Toll-Like Receptor 4-Mediated Nuclear Factor Kappa B Pathway by Lowering Lipopolysaccharide Concentrations

**FIGURE 3:**
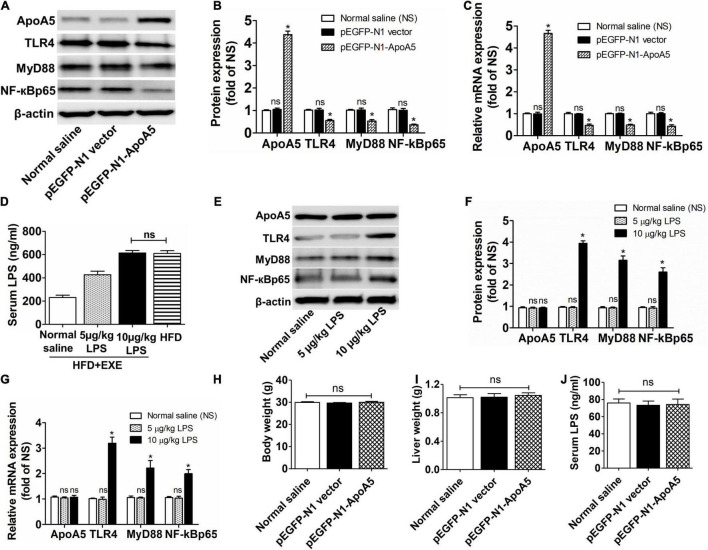
Exercise enhanced the ability of ApoA5 to inhibit TLR4 signaling pathway by lowering LPS concentrations. The hepatic protein **(A,B)** and relative mRNA **(C)** expression levels of ApoA5, TLR4, MyD88, and NF-κBp65 in LFD mice transfected with pEGF-N1-ApoA5. The LPS levels of serum in HFD + EXE mice after injection of different concentrations of LPS **(D)**. The hepatic protein **(E,F)** and relative mRNA **(G)** expression levels of ApoA5, TLR4, MyD88, and NF-κBp65 in HFD + EXE mice after injection of different concentrations of LPS. The body weight **(H)**, liver weight **(I)**, and circulating LPS concentrations **(J)** in LFD mice transfected with pEGF-N1-ApoA5. Results are presented as the mean ± SEM, *n* = 4 per group. * *P* < 0.05 for different from the pEGF-N1 vector group or 5 μg/kg⋅wt group; *^ns^* *P* > 0.05 (no difference) for different from the normal saline group.

To investigate whether ApoA5 could inhibit the TLR4-mediated signaling pathway, we assessed the expression of TLR4, MyD88, and NF-κBp65 after transfection with the ApoA5 overexpression plasmid (pEGF-N1-ApoA5) in LFD mice. Accordingly, LFD mice transfected with pEGF-N1-ApoA5 demonstrated remarkably higher mRNA and protein expression of ApoA5 and distinctly lower mRNA and protein expression of TLR4, MyD88, and NF-κBp65 compared to untransfected mice (Figures 3A–C), indicating that high ApoA5 expression can inhibit the TLR4-mediated NF-κB pathway. However, ApoA5 overexpressed mice unchanged in body weight, liver weight and serum LPS concentrations compared to the untransfected mice (Figures 3H–J).

However, the inhibitory effects of ApoA5 on TLR4 were limited by circulating LPS concentrations. HFD + EXE mice had lower LPS concentrations, which were obviously enhanced after injection of 5 and 10 μg/kg⋅wt of LPS, respectively (Figure 3D). HFD + EXE mice treated with 10 μg/kg⋅wt of LPS had equal LPS concentrations as mice with HFD-induced NASH (614.27 ± 36.21 vs. 609.42 ± 42.21 ng/mL) (Figure 3D). Nevertheless, ApoA5 expression remained unchanged and showed high expression in treated mice (Figures 3E–G). The mRNA and protein levels of TLR4, MyD88, and NF-κBp65 remained unaffected after treatment with 5 μg/kg⋅wt of LPS but were distinctly increased after treatment with 10 μg/kg⋅wt LPS (Figures 3E–G). These results revealed that exercise enhanced the ability of ApoA5 to inhibit TLR4-mediated NF-κB pathway by lowering LPS concentrations.

## Discussion

Non-alcoholic steatohepatitis has been associated with hepatic disease progression, development of cirrhosis, and hepatocellular carcinoma (Ibrahim et al., 2018). The ideal therapy for NASH is one that involves effectively reversing liver injury and fibrosis and improving or at least having no negative effects on other metabolic parameters or cardiovascular comorbidities (Sheka et al., 2020). Lifestyle modifications, such as diet control and exercise, have been the primary treatment for NASH (Goncalves et al., 2013; Neuschwander-Tetri, 2020). Exercise is an important strategy for preventing and treating NASH given its ability to decrease hepatic fat content and insulin resistance, as well as modify *de novo* synthesis of free fatty acids, all of which have an effect on NASH (van der Windt et al., 2018). The present study also confirmed that exercise not only reduced body weight, liver weight, and hepatic lipid accumulation but also attenuated hepatic inflammation and fibrosis in mice with HFD-induced NASH.

Evidence has shown that ApoA5 plays an important role in maintaining plasma TG levels and in the pathogenesis of NAFLD given the association between ApoA5 and storage of TG in intrahepatic lipid droplets (Forte and Ryan, 2015). Similarly, simultaneously increased hepatic TG contents and ApoA5 expression had been detected in our mice with HFD-induced NASH. Studies have observed that patients and mice with NAFLD have elevated levels of hepatic ApoA5 mRNA and protein, which were markedly downregulated after amelioration of hepatosteatosis (Ress et al., 2011; Feng et al., 2015; Lin et al., 2017). Our study also demonstrated that mice with HFD-induced NASH had higher hepatic ApoA5 expression, which remained unchanged after exercise intervention. Interestingly, exercise resulted in a considerable reduction in hepatic TLR4, MyD88, and NF-κBp65 expression and circulating LPS concentrations.

Toll-Like Receptor 4, the main receptor for the recognition of LPS, is upregulated in endotoxin-induced liver injury (Takayashiki et al., 2004). In response to LPS, TLR4 activates the NF-κB pathway to release NF-κBp65 (Medvedev et al., 2000; Byun et al., 2015), which subsequently translocates to the nucleus and stimulates the transcription of inflammatory genes (Li et al., 2014). Therefore, targeting the TLR4-mediated NF-κB pathway may be one method of alleviating hepatic inflammation in NASH. Tao et al. observed that increased ApoA5 expression could attenuate liver injury by inhibiting the TLR4-mediated NF-κB pathway, although the inhibitory effects weakened with increasing LPS concentrations (Tao et al., 2019). Serum LPS levels significantly increased after HFD administration, which were partially reduced after exercise (Yu C. et al., 2019). Based on the results of previous studies and our own, we hypothesized that exercise enhanced the ability of ApoA5 to inhibit the TLR4-mediated NF-κB pathway by lowering LPS concentrations.

To test this hypothesis, we performed the ApoA5 transfection and LPS intervention studies, which showed that increased ApoA5 expression indeed inhibited the expression of TLR4, MyD88, and NF-κBp65—crucial cytokines involved in the TLR4-mediated NF-κB pathway (Figures 2, 3). However, the inhibitory effects of ApoA5 declined when circulating LPS concentrations increased to match those observed in HFD mice (Figure 3D). The current study found that exercise could remarkably reduce serum LPS concentrations while maintaining increased hepatic ApoA5 expression, which triggered the inhibitory effects on the TLR4-mediated NF-κB pathway.

In conclusion, exercise alleviated HFD diet-induced hepatic steatosis, inflammation and fibrosis, all of which were characteristics of NASH. Notably, exercise did not reduce ApoA5 expression but instead increased its ability to suppress TLR4-mediated NF-κB pathway by lowering LPS concentration in mice with HFD-induced NASH. Taken together, our results suggested that exercise may target the ApoA5-TLR4 pathway to improve NASH.

## Data Availability Statement

The original contributions presented in the study are included in the article/supplementary material, further inquiries can be directed to the corresponding authors.

## Ethics Statement

The animal study was reviewed and approved by the Animal Experiment Administration Committee of Guangzhou Sport University (2020DWLL-005).

## Author Contributions

YY and LZ performed study concept and design. YY, LY, XL, and CF performed development of methodology and writing, review, and revision of the manuscript. YY, LY, and SL provided acquisition, analysis and interpretation of data, and statistical analysis. LY provided technical and material support. NC performed supplementary experiments and data analysis. All authors read and approved the final manuscript.

## Conflict of Interest

The authors declare that the research was conducted in the absence of any commercial or financial relationships that could be construed as a potential conflict of interest.

## Publisher’s Note

All claims expressed in this article are solely those of the authors and do not necessarily represent those of their affiliated organizations, or those of the publisher, the editors and the reviewers. Any product that may be evaluated in this article, or claim that may be made by its manufacturer, is not guaranteed or endorsed by the publisher.
